# What Should We Recommend for Colorectal Cancer Screening in Adults Aged 75 and Older?

**DOI:** 10.3390/curroncol28040231

**Published:** 2021-07-09

**Authors:** Anuj Arora, Sami A Chadi, Tyler Chesney

**Affiliations:** 1 Department of Surgery, Division of General Surgery, University of Toronto, University Health Network, Toronto, ON M5T 2S8, Canada; anuj.arora@mail.utoronto.ca (A.A.); sami.chadi@uhn.ca (S.A.C.); 2 Department of Surgery, Division of General Surgery, University of Toronto, Toronto, ON M5N 3M5, Canada; 3 Department of Surgery, Division of General Surgery, St. Michael’s Hospital, Toronto, ON M5B 1W8, Canada; 4 Li Ka Shing Knowledge Institute, St. Michael’s Hospital, Toronto, ON M5B 1T8, Canada

**Keywords:** older adults, colorectal cancer screening

## Abstract

The current recommendation to stop colorectal cancer screening for older adults is based on a lack of evidence due to systematic exclusion of this population from trials. Older adults are a heterogenous population with many available strategies for patient-centered assessment and decision-making. Evolutions in management strategies for colorectal cancer have made safe and effective options available to older adults, and the rationale to screen for treatable disease more reasonably, especially given the aging Canadian population. In this commentary, we review the current screening guidelines and the evidence upon which they were built, the unique considerations for screening older adults, new treatment options, the risks and benefits of increased screening and potential considerations for the new guidelines.

## 1. Are Older Adults Represented in Current Colorectal Cancer Screening Guidelines?

Colorectal cancer screening recommendations in Canada currently recommend not to screen adults aged 75 and older; however, over half of colorectal cancer deaths are expected to occur in patients older than this [1,2]. With longer and healthier living, the number of older adults with colon cancer is rising, comprising the greatest proportion of new colon cancers—now one of the most common cancers in those older than 75 years [1,3,4,5,6]. Considerable efforts have focused on tailoring treatment approaches for older adults with colorectal cancer by considering the heterogeneity of health statuses in older adults highlighted by the concepts of *physiologic age* and *frailty* [7,8,9,10,11]. Furthermore, advancements in minimally invasive surgical and endoscopic techniques offer added benefits for older adults [12,13,14]. Current guidelines do not reflect these important considerations unique to older adults. In fact, most trials investigating colorectal cancer screening have systematically excluded older adults. Here, we discuss how these unique considerations for older adults should be reflected in updated screening guidelines for colorectal cancer. 

## 2. What Evidence Was Used to Generate Current Guideline Recommendations?

Current screening guidelines are based on the absence of a demonstrated benefit in adults aged 75 and older [2]. This absence is not due to a conclusive demonstration that screening lacks benefit in this age group, but rather represents a gap in knowledge due to systematic exclusion of older adults from available studies on which screening recommendations are based (Table 1). No justification is given for the exclusion of older adults. Studies demonstrating benefit of colonoscopy or flexible sigmoidoscopy for screening mostly excluded patients between the ages of 50 and 54, but screening of these patients using these tests is still recommended. The systematic exclusion of older adults is unfortunately common, and strategic recommendations aim to remedy this gap [15,16,17]. Despite the paucity of evidence available to make recommendations due to systematic exclusion of older adults from studies investigating colorectal cancer screening strategies, the authors of the current guidelines chose to recommend not screening in the age group. Other guideline groups have given conditional recommendations to individualize screening decisions but provide little advice on how this should be carried out [18,19,20,21]. 

## 3. What Unique Considerations Are Needed When Assessing Older Adults for Screening?

Recommendations to stop screening beyond age 75 are partly based on reduced life expectancy [2]. Current screening recommendations in older adults do not address the impact of comorbidity, functional status, frailty, and life expectancy, which varies widely among older adults. For example, in a population-based study including 35,755 patients, adults aged 80 or older with no comorbidities had life expectancies greater than 10 years after a diagnosis of stage I colon cancer [33]. Other guidelines specific to the care of older adults recommend older adult-specific assessments to guide shared decision-making [10,11,34,35]. While the comprehensive geriatric assessment is the reference standard process to determine multidimensional health status and develop integrated management plans for older adults, this is not feasible for all older adults and abbreviated screening strategies are advocated [36,37]. This strategy recognizes that an individual’s health status is better understood as multidimensional, including physical, functional, cognitive, and social elements rather than chronologic age alone [7,8]. Many suggested screening tools aim to identify frailty, a state of vulnerability to stressors and increased risk of adverse health outcomes due to multisystem decline in physiologic reserve and function [9,38,39]. Some helpful and commonly used tools that can be used quickly by non-geriatricians include the G8, the clinical frailty scale (CFS), and the vulnerable elders survey-13 (VES-13) [37,40,41,42]. Additionally, decision-support aids are available through ePrognosis (San Francisco, CA, USA), QCancer^®^ (UK), and www.screeningdecision.com (Michigan, MI, USA) (accessed on 1 November 2020) for estimating life expectancy, risk of developing colorectal cancer, and risks and harms of screening [43,44,45,46]. As with the approach suggested for surveillance after polypectomy, this more fulsome assessment of an individual’s health status can be used to appropriately tailor screening decisions through shared decision-making to elicit preferences and values and generate individual screening recommendations [47].

## 4. How Should the Evolution of Treatment Options Impact Screening Recommendations?

Screening recommendations should also consider the availability of safe and effective treatments for an identified disease. Substantial advancements have been made in tailoring treatment approaches for older adults with colorectal cancer [10,11,48]. Advancements include incorporating geriatric-specific assessments and outcomes of importance, along with evidence focused specifically on older adults with colorectal cancer to aid treatment planning. One example is the rising technical capacity and availability of advanced minimally invasive surgery and therapeutic endoscopy [49,50]. These techniques include laparoscopic surgery, transanal local excisions with advanced platforms, and advanced endoscopic techniques including endoscopic submucosal dissection and full thickness resection [51,52,53,54,55,56]. Several studies have shown excellent outcomes for older adults undergoing laparoscopic surgery compared to open surgery, with similar oncologic outcomes [12,13,14,57,58,59]. Similarly, the understanding of nonsurgical treatments alone or in combination with surgery for older adults has grown [60,61,62,63,64,65]. Approaches may include local excision combined with adjuvant treatment, or even the avoidance of surgery all together [66,67,68]. While many older adults may safely undergo standard treatments, several modifications are now available based on an overall multidimensional health assessment to guide treatment choices [69,70,71,72]. With this tailored approach to older adults with colorectal cancer, excellent goal-concordant outcomes can be achieved [73,74]. 

## 5. What Are the Risks and Benefits of Increased Screening?

Certainly, the risks of screening must also be considered. Invasive tests such as colonoscopy appear to have some risk of perforation, bleeding, cardiovascular and pulmonary complications [75]. In contrast, stool-based tests such as fecal immunochemical testing (FIT) are non-invasive and can be safely carried out by older adults [2]. Potential risks of FIT include possible false-positive tests which can increase psychological stress and require further invasive testing [76]. There is potential for overdiagnosis due to a time lag to the benefit of 4.8 years, but this suggests that screening could be continued for older adults with life expectancies greater than this [77]. Patients without screening can present with symptoms such as pain, obstruction, perforation, or bleeding, with much greater risks of treatment in an emergency setting [78,79]. A strategy that carefully balances the risks and benefits of screening based on comprehensive assessment and understanding of tailored treatment approaches is needed.

## 6. What Should Be Considered in New Guidelines?

Considerable work has been carried out to enable individualization of screening recommendations for older adults, but so far, this work has not been included by guideline authors [80,81]. General strategies use age and comorbidities to tailor estimates of life expectancy and the potential benefit of screening [80,82]. Incorporating cancer risk, screening history, and comorbidities could make screening cost-effective [83,84]. This approach can be operationalized with tools such as ePrognosis (San Francisco, CA, USA), QCancer^®^ (UK), and www.screeningdecision.com (Michigan, MI, USA) [43,44,45,46]. Furthermore, communication about prognosis and the cessation of screening should be carefully considered [85,86,87]. Most patients and clinicians prefer the discussion of screening cessation to be framed around overall health status and changing health priorities, while only some feel that an explicit mention of life expectancy was needed using phrases such as “this test would not help you live longer” [88,89]. However, no studies have explored the preferences and values of older adults regarding noninvasive screening and the avoidance of other outcomes such as symptomatic bleeding, obstruction, and emergency surgery. 

## 7. Conclusions

Guideline recommendations for colorectal cancer screening in adults aged 75 and older must incorporate considerations unique to this age group. Recommendations to not screen adults in this age group were generated based on a lack of knowledge resulting from systematic exclusion of older patients from trials. This recommendation is not justified. Considerable advancements in tailoring treatment approaches for this age group such as minimally invasive surgical and endoscopic treatments and nonoperative treatments should be considered in making screening recommendations. A recommendation for individualized screening is much more justified. This recommendation should also contain detailed advice and guidance on individualization including specific assessment of overall health status combined with shared decision-making to generate a recommendation integrating individual preferences and values. Further studies should evaluate strategies to personalize screening recommendations specific to older adults and investigate the outcomes of most importance to them.

## Figures and Tables

**Table 1:** Exclusion of older adults from studies used to inform guidelines about colorectal cancer screening recommendations [2,18,19,21].

Reference	Study Design	Test Modality	Primary Outcome	Ages Included	Mean Age of Included Participants	Rationale for Age Cut Offs
Jorgensen et al., 2002 [22]	RCT	FOBT	Reduction in mortality from CRC	45–75	58.8	None provided
Lindholm et al., 2008 [23]	RCT	FOBT	Reduction in mortality from CRC	60–64	Not provided	None provided
Scholefield et al., 2011 [24]	RCT	FOBT	Reduction in CRC mortality and incidence	45–74	Not provided	None provided
Shaukat et al., 2013 [25]	RCT	FOBT	Reduction in CRC mortality	50–80	62.3 ± 7.8	None provided
Zheng et al., 2003 [26]	RCT	FOBT and quantitative individual risk of colorectal cancer	Reduction in rectal and colon cancer mortality	>30	Mean not provided, 7% of individuals screened were aged 70 and over	Occurrence age of colorectal cancer in Chinese populations is younger than Western populations
Atkin et al., 2010 [27]	RCT	Flexible sigmoidoscopy	Reduction of CRC incidence and mortality	55–64	60 ± 2.9	None provided
Hoff et al., 2009 [28]	RCT	Flexible sigmoidoscopy	Reduction in CRC incidence and mortality	55–64	59	None provided
Schoen et al., 2012 [29]	RCT	Flexible sigmoidoscopy	Reduction in CRC incidence and mortality	55–74	Not provided	None provided
Segnan et al., 2011 [30]	RCT	Flexible sigmoidoscopy	Reduction in CRC incidence and mortality	55–64	59.3 ± 4.4	None provided
Bretthauer et al., 2016 [31]	RCT	Colonoscopy	Participation rate, adenoma yield, performance, adverse events	55–64	60.0 (median)	None provided
Quintero et al., 2012 [32]	RCT	Colonoscopy vs. FIT	CRC specific mortality	50–69	59.2 ± 5.5 in colonoscopy group 59.3 ± 5.6 in FIT group	None provided

RCT—randomized control trial, FOBT—faecal occult blood testing, CRC—colorectal cancer.
